# Supplementary material for: Seasonality and weather dependance of Acinetobacter baumannii complex bloodstream infections in different climates in Brazil
Source: PLoS One. 2021 Aug 19;16(8):e0255593. doi: 10.1371/journal.pone.0255593 (PMC8376013; doi:10.1371/journal.pone.0255593)
Supplement: Attachment — Submitted filename: PONE-D-21-05493_reviewer.pdf [file pone.0255593.s002.pdf]

PLOS ONE

Seasonality and weather dependance of Acinetobacter baumannii complex bloodstream infections in different climates in Brazil.

--Manuscript Draft--

**Manuscript Number:**	PONE-D-21-05493
**Article Type:**	Research Article
**Full Title:**	Seasonality and weather dependance of Acinetobacter baumannii complex bloodstream infections in different climates in Brazil.
**Short Title:**	Acinetobacter baumannii seasonality, Brazil
**Corresponding Author:**	Carlos Magno Castelo Branco Fortaleza, M.D., Ph.D.<br>Universidade Estadual Paulista Julio de Mesquita Filho Faculdade de Medicina Campus de Botucatu<br>Botucatu, São Paulo BRAZIL
**Keywords:**	Acinetobacter baumanni; bloodstream infections; resistance to carbapenems; seasonality
**Abstract:**	Recent studies report seasonality in healthcare-associated infections, especially those caused by Acinetobacter baumannii complex. We conducted a study aimed at analyzing the impact of seasons, weather parameters and climate control on the incidence and carbapenem-resistance in A. baumannii complex bloodstream infections (ABBSI) in hospitals from regions with different climates in Brazil. We studied monthly incidence rates (years 2006-2015) of ABBSI from hospitals in cities from different macro-regions in Brazil: Fortaleza (Ceará State, Northeast region), Goiânia (Goiás State, Middle-west) and Botucatu (São Paulo State, Southeast). Box-Jenkins models were fitted to assess seasonality, and the impact of weather parameters was analyzed in Poisson Regression models. Separate analyses were performed for carbapenem-resistant versus carbapenem-susceptible isolates, as well as for infections occurring in climate-controlled intensive care units (ICUs) versus non-climate-controlled wards. Seasonality was identified for ABSSI ICUs in the Hospitals from Botucatu and Goiânia. In the Botucatu hospital, where there was overall seasonality for both resistance groups, as well as for wards without climate control. In that hospital, the overall incidence was associated with higher temperature (incidence rate ratio for each Celsius degree, 1.05; 95% Confidence Interval, 1.01-1.09; P =0.006). Weather parameters were not associated with ABBSI in the hospitals from Goiânia and Fortaleza. In conclusion, seasonality was found in the hospitals with higher ABBSI incidence and located in regions with greater thermal amplitude. Strict temperature control may be a tool for prevention of A. baumanii infections in healthcare settings.
**Order of Authors:**	Sebastião Pires Ferreira Filho
	Milca Severino Pereira
	Jorge Luiz Nobre Rodrigues
	Raul Borges Guimarães
	Antônio Ribeiro da Cunha
	José Eduardo Corrente
	Antônio Carlos Campos Pignatari
	Carlos Magno Castelo Branco Fortaleza, M.D., Ph.D.
**Additional Information:**	
**Question**	**Response**
**Financial Disclosure**<br><br>Enter a financial disclosure statement that	The authors received no specific funding for this work.

describes the sources of funding for the work included in this submission. Review the submission guidelines for detailed requirements. View published research articles from *PLOS ONE* for specific examples.<br><br>This statement is required for submission and **will appear in the published article** if the submission is accepted. Please make sure it is accurate.<br><br>**Unfunded studies**<br>Enter: *The author(s) received no specific funding for this work.*<br><br>**Funded studies**<br>Enter a statement with the following details:<br>• Initials of the authors who received each award<br>• Grant numbers awarded to each author<br>• The full name of each funder<br>• URL of each funder website<br>• Did the sponsors or funders play any role in the study design, data collection and analysis, decision to publish, or preparation of the manuscript?<br>• **NO** - Include this sentence at the end of your statement: *The funders had no role in study design, data collection and analysis, decision to publish, or preparation of the manuscript.*<br>• **YES** - Specify the role(s) played.<br><br>* typeset	
**Competing Interests**<br><br>Use the instructions below to enter a competing interest statement for this submission. On behalf of all authors, disclose any competing interests that could be perceived to bias this work—acknowledging all financial support and any other relevant financial or non-financial competing interests.<br><br>This statement is required for submission and **will appear in the published article** if the submission is accepted. Please make sure it is accurate and that any funding	The authors have declared that no competing interests exist.

Powered by Editorial Manager® and ProduXion Manager® from Aries Systems Corporation

sources listed in your Funding Information later in the submission form are also declared in your Financial Disclosure statement.<br><br>View published research articles from *PLOS ONE* for specific examples.<br><br>**NO authors have competing interests**<br><br>Enter: *The authors have declared that no competing interests exist.*<br><br>**Authors with competing interests**<br><br>Enter competing interest details beginning with this statement:<br><br>*I have read the journal's policy and the authors of this manuscript have the following competing interests: [insert competing interests here]*<br><br>* typeset	
**Ethics Statement**<br><br>Enter an ethics statement for this submission. This statement is required if the study involved:<br><br>• Human participants<br>• Human specimens or tissue<br>• Vertebrate animals or cephalopods<br>• Vertebrate embryos or tissues<br>• Field research<br><br>Write "N/A" if the submission does not require an ethics statement.<br><br>General guidance is provided below. Consult the submission guidelines for detailed instructions. **Make sure that all information entered here is included in the Methods section of the manuscript.**	This study adheres to the Helsinki declaration guidelines and was approved by the institutions Committees for Ethics in Research (CAAE # 81985517.0.1001.5411).

**Format for specific study types**<br><br>**Human Subject Research (involving human participants and/or tissue)**<br>• Give the name of the institutional review board or ethics committee that approved the study<br>• Include the approval number and/or a statement indicating approval of this research<br>• Indicate the form of consent obtained (written/oral) or the reason that consent was not obtained (e.g. the data were analyzed anonymously)<br><br>**Animal Research (involving vertebrate animals, embryos or tissues)**<br>• Provide the name of the Institutional Animal Care and Use Committee (IACUC) or other relevant ethics board that reviewed the study protocol, and indicate whether they approved this research or granted a formal waiver of ethical approval<br>• Include an approval number if one was obtained<br>• If the study involved *non-human primates*, add *additional details* about animal welfare and steps taken to ameliorate suffering<br>• If anesthesia, euthanasia, or any kind of animal sacrifice is part of the study, include briefly which substances and/or methods were applied<br><br>**Field Research**<br><br>Include the following details if this study involves the collection of plant, animal, or other materials from a natural setting:<br>• Field permit number<br>• Name of the institution or relevant body that granted permission	
**Data Availability**<br><br>Authors are required to make all data underlying the findings described fully available, without restriction, and from the time of publication. PLOS allows rare exceptions to address legal and ethical concerns. See the PLOS Data Policy and FAQ for detailed information.	Yes - all data are fully available without restriction

Powered by Editorial Manager® and ProduXion Manager® from Aries Systems Corporation

A Data Availability Statement describing where the data can be found is required at submission. Your answers to this question constitute the Data Availability Statement and **will be published in the article**, if accepted.<br><br>**Important:** Stating 'data available on request from the author' is not sufficient. If your data are only available upon request, select 'No' for the first question and explain your exceptional situation in the text box.<br><br>Do the authors confirm that all data underlying the findings described in their manuscript are fully available without restriction?	
**Describe where the data may be found in full sentences. If you are copying our sample text, replace any instances of XXX with the appropriate details.**<br><br>• If the data are **held or will be held in a public repository**, include URLs, accession numbers or DOIs. If this information will only be available after acceptance, indicate this by ticking the box below. For example: *All XXX files are available from the XXX database (accession number(s) XXX, XXX.*).<br>• If the data are all contained **within the manuscript and/or Supporting Information files**, enter the following: *All relevant data are within the manuscript and its Supporting Information files.*<br>• If neither of these applies but you are able to provide **details of access elsewhere**, with or without limitations, please do so. For example:<br><br>*Data cannot be shared publicly because of [XXX]. Data are available from the XXX Institutional Data Access / Ethics Committee (contact via XXX) for researchers who meet the criteria for access to confidential data.*<br><br>*The data underlying the results presented in the study are available from (include the name of the third party*	All relevant data are within the manuscript and its Supporting Information files.

and contact information or URL).<br>• This text is appropriate if the data are owned by a third party and authors do not have permission to share the data.<br><br>* typeset	
Additional data availability information:	

Powered by Editorial Manager® and ProduXion Manager® from Aries Systems Corporation

Manuscript

Click here to access/download;Manuscript;renamed_45d50.docx

Title: Seasonality and weather dependance of *Acinetobacter baumannii* complex bloodstream infections in different climates in Brazil.

Short title: *Acinetobacter baumannii* seasonality, Brazil

Authors

Sebastião Pires Ferreira Filho[1], MD, MSc; Milca Severino Pereira[2], RN, PhD; Jorge Luiz Nobre Rodrigues[3], MD, PhD; Raul Borges Guimarães[4], PhD; Antônio Ribeiro da Cunha[5], PhD; José Eduardo Corrente[6], PhD; Antônio Carlos Campos Pignatari[7], MD, PhD; Carlos Magno Castelo Branco Fortaleza, MD[1], PhD

Authors' affiliations

[1]Department of Infectious Diseases, Botucatu School of Medicine, São Paulo State University (UNESP). City of Botucatu, São Paulo State, Brazil.

[2]Depatment of Nursing, Pontifical Catholic University of Goiás (PUCG). City of Goiânia, Goiás State, Brazil.

[3]Center of Health Sciences, Federal University of Ceará (UFC). City of Fortaleza, Ceará State, Brazil.

[4] Department of Geography, Faculty of Sciences and Technology, São Paulo State University (UNESP). City of Presidente Prudente, São Paulo State, Brazil.

[5] Department of Soil and Environmental Resources, Faculty of Agronomical Sciences, São Paulo State University (UNESP). City of Botucatu, São Paulo State, Brazil.

[6] Department of Biostatistics, Botucatu Institute of Biosciences, São Paulo State University (UNESP). City of Botucatu, São Paulo State, Brazil.

[7] Department of Medicine, São Paulo Federal University (UNIFESP). City of São Paulo, São Paulo State, Brazil.

Corresponding author

Carlos Magno Castelo Branco Fortaleza
Departamento de Doenças Infecciosas, Faculdade de Medicina de Botucatu
Street address: Avenue Professor Mário Rubens Guimarães Montenegro, s/n
City of Botucatu, São Paulo State, Brazil
CEP/ZIP CODE: 18618-687
Phone: +55 14 3880 1284
Email: carlos.fortaleza@unesp.br

Declaration of interest: NONE

Abstract

Recent studies report seasonality in healthcare-associated infections, especially those caused by *Acinetobacter baumannii* complex. We conducted a study aimed at analyzing the impact of seasons, weather parameters and climate control on the incidence and carbapenem-resistance in *A. baumannii* complex bloodstream infections (ABBSI) in hospitals from regions with different climates in Brazil. We studied monthly incidence rates (years 2006-2015) of ABBSI from hospitals in cities from different macro-regions in Brazil: Fortaleza (Ceará State, Northeast region), Goiânia (Goiás State, Middle-west) and Botucatu (São Paulo State, Southeast). Box-Jenkins models were fitted to assess seasonality, and the impact of weather parameters was analyzed in Poisson Regression models. Separate analyses were performed for carbapenem-resistant versus carbapenem-susceptible isolates, as well as for infections occurring in climate-controlled intensive care units (ICUs) versus non-climate-controlled wards. Seasonality was identified for ABSSI ICUs in the Hospitals from Botucatu and Goiânia. In the Botucatu hospital, where there was overall seasonality for both resistance groups, as well as for wards without climate control. In that hospital, the overall incidence was associated with higher temperature (incidence rate ratio for each Celsius degree, 1.05; 95% Confidence Interval, 1.01-1.09; *P*=0.006). Weather parameters were not associated with ABBSI in the hospitals from Goiânia and Fortaleza. In conclusion, seasonality was found in the hospitals with higher ABBSI incidence and located in regions with greater thermal amplitude. Strict temperature control may be a tool for prevention of *A. baumanii* infections in healthcare settings.

Keywords

Acinetobacter baumanni; bloodstream infections; resistance to carbapenems; seasonality

Introduction

Despite their specific characteristics, healthcare-associated infections (HCAIs) can share some epidemiological determinants with those that occur in the community. Seasonality, increasingly identified in recent studies, is one example [1]. It is prominent in bloodstream infections caused by Gram-negative bacilli (GNB), which have been linked to proximity to the equator [2], summer season [3] and high environmental temperatures measured either within [4] or outside hospitals [5].

This latter aspect is one of the gaps in our current understanding of HCAIs seasonality, since GNB incidence increases during warm periods even within units that are climate-controlled (and thus expected not to present relevant temperature variations) [1,5,6,7]. Some authors have theorized an influx from reservoirs outside healthcare settings, on the basis of greater seasonality of multidrug-susceptible (as opposed to multidrug resistant, supposedly "hospital-borne") GNB [8] or on molecular heterogeneity of summer strains (which suggests multiple sources) [9]. However, those findings were not supported by other studies, especially those pointing to relevant "summer peaks" of multidrug-resistant GNB infections [6,7,10].

Acinetobacter baumannii complex bloodstream infections (ABBSI) stand out among GNB HCAIs for their striking seasonal pattern [11]. They pose therefore unique opportunities to assess HCAIs seasonality. With that in mind, we conducted an ecological study aimed at analyzing how that seasonality of ABBSI varies in different climates, as well as its association with antimicrobial resistance and climate control in hospital units.

Methods

Ethical statement

This study adheres to the Helsinki declaration guidelines and was approved by the institutions Committees for Ethics in Research (CAAE # 81985517.0.1001.5411).

Study settings.

This study was conducted in three hospitals from cities located in different macro-regions of Brazil: Fortaleza (Ceará State, Northeast region), Goiânia (Goiás State, Middle-west) and Botucatu (São Paulo State, Southeast). In all hospitals, Intensive Care Units (ICUs) were climate-controlled, while other wards were not climatized. **Figure 1** presents the geographic distribution of those cities within the map of Brazil. Geographic and climate aspects of those cities are presented in **Table 1**. The study cities differed from each other in the Köppen-Geiger climate classification, according to the Brazil's "National Institute for Spatial Research" (INPE, www.inpe.br) [12].

FIGURE UPLOADED IN A SEPPARATE FILE

Figure 1. Map of Brazil, showing the cities that host the study hospitals.

Table 1. Climatic and geographic characteristics of the municipalities where the study hospitals are located.

Hospital	Botucatu Medical School Hospital	Goiás Federal University Hospital	Ceará Federal University Hospital
City	Botucatu	Goiânia	Fortaleza
State	São Paulo	Goiás	Ceará
Macro-region	Southeast	Middle-west	Northeast
Latitude, longitude	22°53′25″S, 48°27′19″W	16°40'48''S, 49°15'18''W	3°43'6''S, 38°32'36''W
Altitude (meters above sea level)	828	764	14
Geographic distance to the Atlantic Ocean (in Kilometers)	246	868	0
Köppen-Geiger climate classification*	*Cfa*	*Aw*	*As*
Mean monthly temperature in Celsius degrees, average (SE)	22.3±3.4	23.5±1.5	27.4±0.7
Mean monthly relative humidity, average (SE)	72.7±8.7	65.8±12.5	71.9±6.1
Monthly total rainfall in millimeters, average (SE)	119.4±100.6	120.4±110.7	103.2±124.9

**Biomes****	Atlantic forest	"Cerrado"	Mangrove/Sandbank

*According to Köppen-Geiger classification[11]: *Cfa*, subtropical climate, humid; *Aw*, tropical climate with dry winter; *As*, tropical with dry summer.

**According to the classification of Brazilian Institute for Geography and Statistics (IBGE, www.ibge.gov.br)

Study data

Monthly incidence rates of ABBSI (per 10,000 patient-days) were calculated for each hospital. Separate rates were calculated for carbapenem-susceptible and carbapenem-resistant *A. baumannii*, as well as for units with or without climate control. Weather parameters (monthly average temperature and humidity, monthly aggregated rainfall) were obtained from INPE climate database.

Time series analysis

Incidence rate series were fitted to stochastic, Box-Jenkins models (Seasonal Autoregressive Integrated Moving Average [SARIMA]) [13]. Those models allowed us to assess statistically the correlations both with data from immediately preceding months and seasons in preceding years. Briefly, models were configured in the usual SARIMA(p,d,q)(P,D,Q)m format using the following trend elements: trend autoregression order (p), 1; trend difference order (d), 0; trend moving average order (q), 0; seasonal autoregressive order (P), 1; seasonal difference order (D), 0; seasonal moving average order (Q), 0; number of time steps (m), 12. Therefore, we attempted to fit our times series to the models parameters: SARIMA (1,0,0)(1,0,0)12.

Models of weather dependence

Multivariable Poisson regression models were analyzed with average monthly temperature and humidity, as well as aggregate monthly rainfall as independent

variables and monthly rates as outcomes. Both Poisson regression an time series analyses were conducted using STATA 14 software (StataCorp, College Station, TX).

Results

The aggregate incidence of ABSSI in the study period (for each type of hospital unit and pattern of carbapenem-resistance) is presented in **Table 2**. Briefly, the Botucatu hospital presented higher overall incidence, but also a greatest proportion of carbapenem-susceptible isolates (especially in years before 2011), when compared to the other two hospitals.

Table 2. Characteristics of hospitals included in this study, alongside with aggregate incidence of *Acinetobacter baumanii* bloodstream infections (per 10,000 patient-days).

Hospital	Botucatu Medical School Hospital	Goiás Federal University Hospital	Ceará Federal University Hospital
Teaching hospital	Yes	Yes	Yes
Beds in climate-controlled units	98	86	21
Beds in non-climate-controlled units	462	634	165
ABBSI, climate-controlled units*			
Carbapenem-susceptible	10.20	1.51	0.56
Carbepenem-resistant	12.96	16.60	6.88
ABBSI, non-climate-controlled units*			
Carbapenem-susceptible	1.27	1.83	0.34
Carbepenem-resistant	1.30	3.00	0.87

*In all study hospitals, Intensive Care Units (ICU) were climate-controlled, while other others were not. ABBSI, *Acinetobacter baumannii* complex bloodstream infections.

Results from the Box-Jenkins models are presented in **Table 3**. Briefly seasonality was identified for ABSSI in ICUs in the Hospitals from Botucatu and Goiânia. In the Botucatu hospital, there was also overall seasonality for both resistance groups, as well as for wards without climate control.

Table 3. Seasonal autoregressive coefficients from Box-Jenkins models for incidence of *Acinetobacter baumannii* complex bloodstream infections, according to type of hospital unit and resistance

	Autoregressive coefficient (95% Confidence Interval)		
**Hospital Unit, carbapenem suscetibility**	**Botucatu**	**Goiânia**	**Fortaleza**
ICU, carbapenem-susceptible	**+0.24 (+0.06 to +0.42)**	-0.03 (-11.34 to +11.29)	+0.09 (-0.05 to +0.23)
ICU, carbapenem-resistant	+0.19 (-0.01 to +0.39)	**+0.32 (+0.21 to +0.43)**	+0.09 (-0.05 to +0.22)
ICU, total	**+0.25 (+0.09 to +0.41)**	**+0.34 (+0.24 to +0.44)**	+0.09 (- 0.05 to +0.23)
Wards, carbapenem-susceptible	-0.01 (-0.16 to +0.17)	+ 0.08 (-0.10 to +0.25)	+0.06 (-0.12 to +0.25)
Wards, carbapenem-resistant	+0.14 (-0.04 to +0.33)	- 0.06 (-0.42 to + 0.31)	-0.12 (-0.22 to +0.21)
Wards, total	**+0.27 (+0.12 to +0.41)**	+0.04 (-0.14 to +0.23)	+0.07 (-0.14 to +0.28)
Overall ABBSI	**+0.26 (+0.11 to +0.41)**	+ 0.14 (-0.06 to +0.33)	+0.08 (-0.10 to +0.26)

Note. Time series were tested for fitting a Box Jenkins Seasonal Autoregressive Moving Average (SARIMA) model. Statistically significant results (P<.05) are presented in boldface. All Intensive Care Units (ICU) were climate controlled, while other wards were not.

In the Poisson regression analysis (**Table 4**), we found significant association of temperature with carbapenem-resistant and overall ABBSI in non-climate-controlled units in the Botucatu hospital. There was also a similar association when we used overall hospital incidence of ABBSI as outcome. No association with weather was found for the other study hospitals.

Table 4. Poisson regression analysis of the impact of weather on monthly incidence of Acinetobacter baumannii complex bloodstream infections, according to type of hospital unit and resistance.

Type of unit / Carbapenem-resistance	Average temperature (ºC)		Average relative Humidity (%)		Total rainfall	
	IRR (95%CI)	*P*	RR (IC95CI%)	*P*	IRR (95%CI)	*P*
Botucatu hospital						
ICU, carbapenem-susceptibe	1.06 (0.99-1.12)	0.08	0.99 (0.96-1.03)	.96	0.99 (0.99-1.01)	0.22
ICU, carbapenem-resistant	1.01 (0.95-1.07)	0.86	0.99 (0.96-1.02)	.48	0.99 (0.99-1.00)	0.97
ICU, total ABBSI	1.03 (0.99-1.08)	0.19	0.99 (0.97-1.01)	.57	0.99 (0.99-1.00)	0.39
Wards, carbapenem-susceptible	1.03 (0.96-1.11)	0.43	1.00 (0.96-1.04)	.84	1.00 (0.99-1.01)	0.87
Wards, carbapenem-resistant	**1.07 (1.01-1.15)**	**0.04**	1.00 (0.96-1.04)	.99	0.99 (0.99-1.00)	0.78
Wards, total ABBSI	**1.05 (1.01-1.10)**	**0.04**	1.00 (0.98-1.03)	.81	0.99 (0.99-1.00)	0.89
Overall ABBSI	**1.05 (1.01-1.09)**	**0.006**	0.99 (0.98-1.01)	.32	0.99 (0.99-1.00)	0.85
Goiânia hospital						
ICU, carbapenem-susceptibe	2.25 (0.96-5.26)	0.06	1.11 (0.96-1.28)	.15	0.98 (0.96-1.03)	0.09
ICU, carbapenem-resistant	1.01 (0.79-1.28)	0.96	1.02 (0.98-1.07)	.29	0.99 (0.99-1.00)	0.24
ICU, total ABBSI	1.08 (0.86-1.36)	0.48	1.03 (0.99-1.07)	.17	0.99 (0.98-1.00)	0.09
Wards, carbapenem-susceptible	1.23 (0.82-1.87)	0.31	0.96 (0.89-1.02)	.16	1.01 (0.99-1.02)	0.06
Wards, carbapenem-resistant	0.79 (0.57-1.08)	0.14	0.99 (0.93-1.04)	.61	1.00 (0.99-1.01)	0.33

Wards, total ABBSI	0.92 (0.73-1.18)	0.54	0.97 (0.93-1.01)	0.17	1.01 (0.99-1.01)	0.09
Overall ABBSI	1.00 (0.84-1.17)	0.97	0.99 (0.97-1.03)	0.97	1.00 (0.99-1.00)	0.89
Fortaleza hospital						
ICU, carbapenem-susceptibe	0.67 (0.11-4.02)	0.67	0.86 (0.62-1.21)	0.41	0.99 (0.97-1.02)	0.51
ICU, carbapenem-resistant	1.27 (0.79-20.07)	0.32	0.98 (0.89-1.08)	0.72	0.99 (0.99-1.00)	0.74
ICU, total ABBSI	1.21 (0.77-1.93)	0.41	0.98 (0.89-1.06)	0.57	0.99 (0.99-1.00)	0.67
Wards, carbapenem-susceptible	1.28 (0.57-2.85)	0.55	0.97 (0.86-1.08)	0.56	1.00 (0.99-1.01)	0.31
Wards, carbapenem-resistant	1.17 (0.74-1.88)	0.49	0.99 (0.91-1.08)	0.88	0.99 (0.99-1.01)	0.81
Wards, total ABBSI	1.19 (0.80-1.79)	0.38	0.98 (0.91-1.06)	0.64	1.00 (0.99-1.01)	0.75
Overall ABBSI	1.20 (0.88-1.63)	0.24	0.98 (0.93-1.03)	0.45	0.99 (0.99-1.00)	0.99

Note. Results with $P<0.05$ are presented in boldface. ABSSI, *Acinetobacter baumannii* complex bloodstream infections; ICU, intensive care units; IRR, incidence rate ratio.

Discussion

Brazil is a huge country with an area of 8.5 million square kilometers and 200 million inhabitants, distributed 27 states located in five macro-regions. Climates range from equatorial in the Amazon basin to temperate in the South region. There are great socioeconomic differences, with poorer areas in the North/Northeast and more developed states in the South/Southeast [14]. Some community-associated infectious diseases are restricted to a climate or biome (e.g., malaria in Amazon), while other occur seasonally in most areas (e.g., Dengue fever) [15]. Among all this complexity, one may wonder how geography and climate impact on the epidemiology of HCAIs occurring in circa 6,000 hospitals in Brazil.

A. baumannii complex infections are hyperendemic in Brazilian hospitals [16]. As in other countries, they preferentially affect critically ill patients, undergoing invasive procedures or carrying invasive devices [11,17]. Our study aimed, therefore, at comparing the impact of season and weather on ABBSI incidence in areas that were both geographically distant and presented different climates.

Counterintuitively, ABBSI incidence was higher in the hospital located in the area presenting lower temperatures [2]. Two aspects highlight differences in the epidemiology of ABBSI in the Botucatu hospital. First, continuous incidence is detected, while in other hospitals there were gaps during which *A. baumannii* was not recovered from cultures. (Second, as already mentioned, there was hyperendemicity of carbapenem-susceptible isolates up to year 2010, while in other

hospitals more than 90% of isolates recovered all through the study period were carbapenem-resistant.

Seasonality results did not exactly match findings from Poisson regression analysis, using weather parameters as predictors. Some aspects may account for those findings. First, one must notice that weather data were obtained from meteorological stations, located in the same city of (but not inside or nearby) the study hospitals. Therefore, they do not necessarily reflect, for instance, temperature and humidity inside hospital wards. This difference is obviously more relevant for the climate-controlled ICUs, though previous studies from our group have found association of temperature outside hospitals with ABBSI incidence in those units [5,6].

Another confounding aspect is the overall difference in the incidence of ABBSI among study hospitals. As one may infer from the Box-Jenkins models presented in **Table 3**, lower incidences hindered subgroup analyses and may have impacted on statistical power of regression models. Also, the variation of temperature among months (and seasons) was greater in the Botucatu (standard error [SE], 3.3) than in Goiânia (SE, 1.5) and Fortaleza (SE, 0.7). That means that the lower the latitude, the lesser the temperature changes though the year.

Finally, there are potential drivers of seasonality which are not directly associated with weather, such summer understaffing [1], the presence of new students and resident doctors or even changes in diseases leading to hospital admission. All those factors require further investigation, though a previous study found no impact of patients' severity-of-illness or comorbidities on the seasonality of Gram-negative (including *A. baumannii*) bloodstream infections [7].

Our study was limited for not individually assessing the average severity-of-illness of patients admitted to different hospitals, as well as their structure for infection control and the quality of microbiology laboratory. However, the three hospitals were included in a recent multistate survey, with similar performance [18]. The same study found minor differences on overall HCAIs incidence among different regions in Brazil [19]. Other limitation was not assessing the incidence of pathogens (e.g., MRSA and GNB) that may compete ecologically for human and inanimate reservoirs inside hospitals [20]. It is therefore possible that the greater incidence of GNB such as *Klebsiella* spp in hospitals close to the Equator line[2] can impact on the lower incidence of *A. baumannii* found in our study.

In conclusion, we found seasonality of *A. baumannii* infections in hospitals located in areas with climates ranging from tropical to temperate. Both incidence, seasonality and association with weather were greater in the hospital located in the area with fresher climate and greater temperature range. Besides reinforcing the seasonal nature of that pathogen, this study points to air conditioning in hospital wards (which is uncommon in low-to-middle income countries [21]) as a tool for infection control and prevention.

Ackowledgement

This study is part of the PhD Thesis of SPFF, with CMCBF as his advisor.

Conflict of interest

All the authors state that they have no conflict of interest regarding this study.

Funding

There was no funding for this study.

References

1. Richet H. Seasonality in Gram-negative and healthcare-associated infections. Clin Microbiol Infect 2012;18:934-40.
2. Fisman D, Patrozou E, Carmeli Y, Perencevich E, Tuite AR, Mermel LA; Geographical Variability of Bacteremia Study Group. Geographical variability in the likelihood of bloodstream infections due to gram-negative bacteria: correlation with proximity to the equator and health care expenditure. PLoS One 2014;9: e114548.
3. Anderson DJ, Richet H, Chen LF, Spelman DW, Hung YJ, Huang AT, Sexton DJ, Raoult D. Seasonal variation in *Klebsiella pneumoniae* bloodstream infection on 4 continents. J Infect Dis 2008;197:752-6.
4. Limaylla DC, Fortaleza CMCB, Silva MO. Environmental conditions and health care-associated infections in wards for noncritical patients. Am J Infect Control 2019;47:599-600.
5. Schwab F, Gastmeier P, Meyer E. The warmer the weather, the more gram-negative bacteria - impact of temperature on clinical isolates in intensive care units. PLoS One 2014;9:e91105.
6. da Silveira M, da Silva LP, de Oliveira Emídio ZP, Fortaleza CMCB. Weather, climate control, and imipenem-resistance in *Acinetobacter baumannii*: an ecological approach. Infect Control Hosp Epidemiol 2018;39:1133-1135.

7. Rodrigues FS, Clemente de Luca FA, Ribeiro da Cunha A, Fortaleza CMCB. Season, weather and predictors of healthcare-associated Gram-negative bloodstream infections: a case-only study. J Hosp Infect 2019;101:134-141.
8. Fukuta Y, Clarke LG, Shields RK, Wagener MM, Pasculle AW, Doi Y. Lack of seasonality in the occurrence of multidrug-resistant *Acinetobacter baumannii* complex. Infect Control Hosp Epidemiol 2012;33:1051-2.
9. Christie C, Mazon D, Hierholzer W Jr, Patterson JE. Molecular heterogeneity of *Acinetobacter baumannii* isolates during seasonal increase in prevalence. Infect Control Hosp Epidemiol 1995;16:590-4.
10. Eber MR, Shardell M, Schweizer ML, Laxminarayan R, Perencevich EN. Seasonal and temperature-associated increases in gram-negative bacterial bloodstream infections among hospitalized patients. PLoS One 2011;6:e25298.
11. Kritsotakis EI, Groves-Kozhageldiyeva A. A systematic review of the global seasonality of infections caused by *Acinetobacter* species in hospitalized patients. Clin Microbiol Infect 2020;26:553-562.
12. Beck HE, Zimmermann NE, McVicar TR, Vergopolan N, Berg A, Wood EF. Present and future Köppen-Geiger climate classification maps at 1-km resolution. Sci Data 2018;5:180214.
13. Box GE, Jenkins GM, Reinsel GC, Ljung GM. Time Series Analysis: Forecasting and Control. Hoboken NJ: Wiley, 2015.
14. Barrozo LV, Fornaciali M, de André CDS, et al. GeoSES: A socioeconomic index for health and social research in Brazil. PLoS One 2020;15:e0232074.
15. Ellwanger JH, Kulmann-Leal B, Kaminski VL, Valverde-Villegas JM, Veiga ABGD, Spilki FR, Fearnside PM, et al. Beyond diversity loss and climate

change: Impacts of Amazon deforestation on infectious diseases and public health. An Acad Bras Cienc 2020;92:e20191375.

16. Labarca JA, Salles MJ, Seas C, Guzmán-Blanco M. Carbapenem resistance in Pseudomonas aeruginosa and *Acinetobacter baumannii* in the nosocomial setting in Latin America. Crit Rev Microbiol 2016;42:276-92.
17. Castelo Branco Fortaleza CM, Moreira de Freitas F, da Paz Lauterbach G. Colonization pressure and risk factors for acquisition of imipenem-resistant *Acinetobacter baumannii* in a medical surgical intensive care unit in Brazil. Am J Infect Control 2013;41:263-5.0
18. Padoveze MC, Fortaleza CM, Kiffer C, Barth AL, Carneiro IC, Giamberardino HI, et al. Structure for prevention of health care-associated infections in Brazilian hospitals: A countrywide study. Am J Infect Control 2016;44:74-9.
19. Fortaleza CMCB, Padoveze MC, Kiffer CRV, Barth AL, Carneiro ICDRS, Giamberardino HIG, et al. Multi-state survey of healthcare-associated infections in acute care hospitals in Brazil. J Hosp Infect 2017;96:139-144.
20. Lastoria LC, Caldeira SM, Moreira RG, Akazawa RT, Maion JC, Fortaleza CM. Ecological competition and the incidence of *Acinetobacter baumannii* bloodstream infections in a teaching hospital in Southeastern Brazil. Rev Soc Bras Med Trop 2014;47:583-8.
21. Limaylla DC, Silva MO, Fortaleza CMCB. Temperature, humidity, and climate control in hospital units: A clue for understanding the seasonality of healthcare-associated pathogens. Infect Control Hosp Epidemiol 2019;40:829-830.

Figure 1 - tiff

Click here to access/download;Figure;PLoS_Figure 1.tif

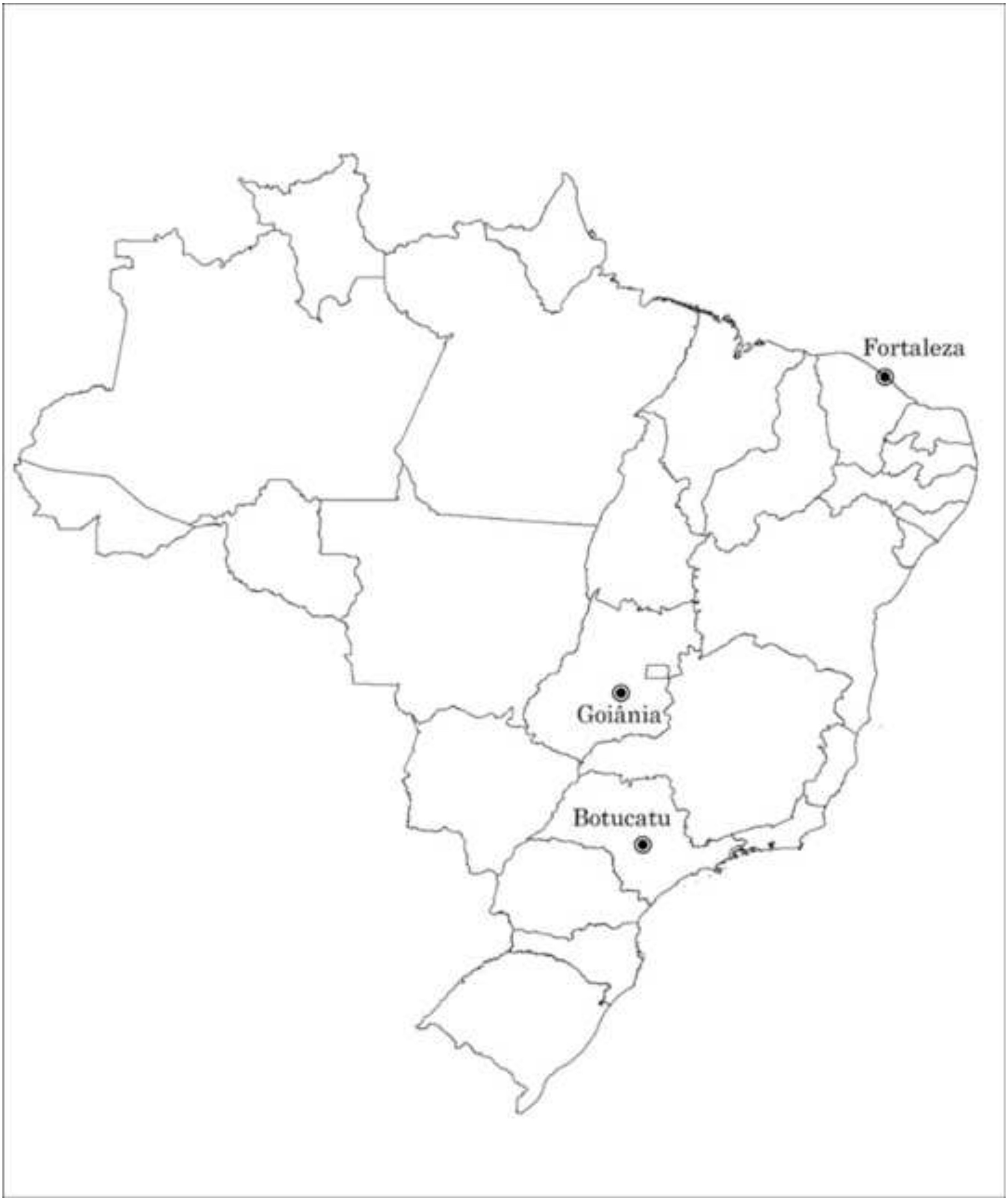

Click here to access/download
Supporting Information - Compressed/ZIP File Archive
StudyData.rar
