# Supplementary material for: Seasonality and weather dependance of Acinetobacter baumannii complex bloodstream infections in different climates in Brazil
Source: PLoS One. 2021 Aug 19;16(8):e0255593. doi: 10.1371/journal.pone.0255593 (PMC8376013; doi:10.1371/journal.pone.0255593)
Supplement: Attachment — Submitted filename: PONE-review.docx [file pone.0255593.s003.docx]

**PONE-D-21-05493**

**Report**

In this manuscript the authors analyzed the seasonal changes of ABBSI in different climates, and its relationship with antibiotic resistance and climate control in hospitals. The manuscript seems scientifically sound, and contains some interesting results that can be considered for publication in PONE. However, before the decision of acceptance for publication is running, a very major revison of the manuscript is required. Specifically, the following points should be addressed by the authors:

1、In general, there is a lack of explanation of replicates and statistical methods used in the study. It is expected that the author can give a more detailed explanation or basis why SARIMA is selected in the time series analysis and multivariate Poisson regression models, is selected in the weather-dependent model.

2、Another major question needs to be explained by the author: why only three hospitals are selected, and is the argument for the conclusion sufficient? In addition, whether it is possible to increase the number of hospital cases selected in the same city (that is, under the same climatic conditions) to see if more interesting results can be obtained, and further confirm the conclusion.
